# miR-203, fine-tunning neuroinflammation by juggling different components of NF‐κB signaling

**DOI:** 10.1186/s12974-022-02451-9

**Published:** 2022-04-12

**Authors:** Shufang Li, Linpeng Li, Jieli Li, Xiaosheng Liang, Chao Song, Yi Zou

**Affiliations:** 1 The Key Laboratory of Virology of Guangzhou, Jinan University, Guangzhou, China; 2 Department of Biology, School of Life Science and Technology, Jinan University, Guangzhou, China

**Keywords:** Neuroinflammation, miR-203, Akirin2, 14-3-3θ, NF‐κB

## Abstract

**Background:**

miR-203 was first indicated in maintaining skin homeostasis and innate immunity. Aberrant expression of miR-203 was found associated with pathological progressions of immune disorders, cancers, as well as neurodegenerations. Recently, increasing data on miR-203 in regulating neuroinflammation and neuronal apoptosis has raised extensive concern about the biological function of this microRNA.

**Methods:**

Mouse model with ectopic miR-203 expression in the hippocampus was constructed by stereotactic injection of lentiviral expression vector of pre-miR-203. Association of miR-203 and mRNA of Akirin2, as well as the competition for miR-203 targeting between Akirin2 3ʹUTR and another recently characterized miR-203 target, 14-3-3θ, was verified using Dual-Luciferase Reporter Gene Assay and western blot. Microglia activation and pro-inflammatory cytokines expression in the hippocampus of mice overexpressing miR-203 was evaluated using immunohistochemistry analysis and western blot. Neuronal cell death was monitored using anti-caspase 8 in immunohistochemistry as well as TUNEL assay. Cognition of mice was assessed with a behavior test battery consisting of nesting behavior test, Barnes maze and fear conditioning test.

**Results:**

Akirin2, an activator of NF‐κB signaling, was identified as a direct target of miR-203. By also targeting 14-3-3θ, a negative regulator of NF‐κB signaling, miR-203 displayed an overall pro-inflammatory role both in vitro and in vivo. Promoted nuclear translocation of NF‐κB and increased expression of proinflammatory cytokines were observed in cultured BV2 cells transfected with miR-203 mimics. Microglia activation and upregulation of NF‐κB, IL-1β and IL-6 were observed in mouse hippocampus with overexpression of miR-203. In addition, promoted neuronal cell death in the hippocampus and impaired neuronal activities resulted in cognitive dysfunction of mice with ectopic miR-203 expression in the hippocampus.

**Conclusion:**

A pro-inflammatory and neurodisruptive role of miR-203 was addressed based on our data in this study. Given the identification of Akirin2 as a direct target of miR-203 and the competition with 14-3-3θ for miR-203 targeting, together with the findings of other signaling molecules in NF‐κB pathway as targets of miR-203, we proposed that miR-203 was a master modulator, fine-tunning neuroinflammation by juggling different components of NF‐κB signaling.

**Supplementary Information:**

The online version contains supplementary material available at 10.1186/s12974-022-02451-9.

## Introduction

Neuroinflammation is an immune reaction in responding to a variety of endogenous and exogenous factors, such as aberrant protein synthesis, increased oxidative stress and pathogen infection, etc. The intensity and the duration of neuroinflammation depend on the cause and context and is a part of the systemic immune response. A well-controlled inflammatory response is essential for pathogen elimination and damage repairing. However, sustained inflammatory cytokine release and the production of reactive oxygen species (ROS) eventually lead to a prolonged and exaggerated inflammation, which is destructive and is one of the common pathologies underlining traumatic CNS injury, aging, as well as neurodegeneration, such as Alzheimer’s disease (AD), Pakinson’s disease (PD), and depression [1]. Microglia, the resident innate immune cells in CNS, usually act earlier than other glial cells and play a pivotal role in physiological immune surveillance as well as in pathological progression of neuroinflammation [2]. In general, a subset of microglia cells (M2) is activated even in the resting state of CNS and low level anti-inflammatory cytokines such as IL-4, IL-10 and TGF-β are released, which are essential for immune-to-brain communication [3]. Conversely, the harmful stimuli including pathogen-associated molecular patterns (PAMPs) and damage-associated molecular patterns (DAMPs) are recognized by pattern recognition receptors (PRRs) such as Toll-like receptors (TLR) on microglia (M1 subtype) and activate NF‐κB signaling to promote microglial phagocytosis and release pro-inflammatory mediators [4]. Pro-inflammatory factors (TNF-α, IL-6, IL-1β, IFN-γ, NO, and ROS etc.) in turn activate NF‐κB signaling via cytokine receptors and upregulate the expression of the later, which result in a positive feedback enhancement of the inflammation [5]. Therefore, precisely regulated production of pro- and anti-inflammatory cytokines is necessary for appropriate immune reactions that elicit neural protective effects.

NF‐κB was originally identified as a B-lymphocyte cell-specific pleiotropic transcription factor, consisting of Rel protein dimers that regulate inducible expression of inflammatory response genes [6]. Upon DAMPs/PAMPs binding, activated TLRs on microglia recruit myeloid differentiation primary response 88 (MYD88) and subsequently recruit inhibitor of κB kinase (IKK) [7]. As a consequence of IKK activation, the inhibitor protein IκB is phosphorylated/degraded and dissociated from NF‐κB, which subsequently translocates to nucleus and lead to increased transcription of pro-inflammatory factors. Given the critical role it played in regulating immune response and other cell activities, converging evidence suggested that NF‐κB signaling pathway was modulated by protein activators/inhibitors as well as by miRNAs [8]. For example, the expression of miR-146 and miR-155 are negatively correlated with NF‐κB activation and both microRNAs directly target key modulators including IRAK1, TRAF6, and IKK to downregulate NF‐κB pathway [8–10]. This negative feedback regulation of NF‐κB pathway is important for a balanced immune activity and dysregulated expression of these miRNAs have been implicated in inflammatory neuropathology underpinning neural degeneration as well as psychological disorders [11, 12].

miR-203 has recently been identified as a hub miRNA in regulating neuron activity in CNS [13]. Upregulated miR-203 was found in the frontal cortex of frontotemporal dementia (FTD) mouse models and caused disease phenotype-associated neuronal cell death. Further investigations revealed multiple direct targets of miR-203, such as Bcl2l2, Dgkb, Mapk10 and Vsnl1 etc., involving in apoptotic pathways. On the other hand, miR-203 was also found to target multiple factors involved in the NF‐κB-dependent inflammatory pathways, such as IL-24, IL-8, MyD88, and SOCS3 in peripheral tissues as well as in CNS [14, 15].

Here, in this study, we presented a new protein target in CNS of miR-203, Akirin2, an evolutionarily conserved nuclear protein that regulate target gene expression via bridging transcription factors and chromatin remodeling complexes, such as Brahma (SWI/SNF) ATP-dependent chromatin-remodeling complex [16]. Direct interaction has been displayed between Akirin2 and the p50 subunit of NF‐κB, which translocate to the nucleus as a complex of NF‐κB-Akrin2-SWI/SNF to activate pro-inflammatory gene expression. Competition for binding of miR-203 was displayed between Akirin2 and 14-3-3θ, a negative regulator of NF‐κB signaling and also a protein target of miR-203 in CNS verified in our recent research [17]. An overall pro-inflammatory role of miR-203 was evidenced by the activated NF‐κB signaling and elevated expression of pro-inflammatory factors in cultured cells transfected with miR-203 mimics, as well as in vivo in mouse hippocampus overexpressing miR-203. In addition to target protein factors in apoptotic pathway, we proposed that the increased neuronal cell death observed in the hippocampus of mice overexpressing miR-203 could at least partly be the consequences of activated microglia and excessive neuroinflammation. The hippocampus-related cognitive dysfunction was further demonstrated in mice with ectopic miR-203 overexpression in the hippocampus using behavior assessment. Given the multiple NF‐κB signaling molecules identified as direct protein targets of miR-203, we propose that miR-203 play a central role in regulating inflammatory activity in CNS via NF‐κB signaling pathway and has a great potential to serve as a therapeutic target to alleviate neuroinflammation, which underpinning the progression of various acute and chronic neurological damages.

## Materials and methods

### Animals

Wild type C57BL/6J mice (male, 4-month-old, 20–25 g) were purchased from The Experimental Animal Center of Guangdong (Guangzhou, China). All mice were housed in the Laboratory Animal Management Center of the Jinan University in a pathogen free facility. All procedures involving animals was approved by the Institutional Animal Care and Use Committee of Jinan University (Approval No. 20201106-01) and were performed in accordance with the animal research committee of Jinan University and the National Institutes of Health (NIH) *Guide for the Care and Use of Laboratory Animals*.

### Reagents

DMEM and fetal bovine serum (FBS) used in cell culture were purchased from Invitrogen. 3,3-diaminobenzidine tetrachloride (DAB), 1,4-dithiothreitol (DTT), dimethyl sulfoxide (DMSO), Triton X-100, 4% paraformaldehyde, bovine serum albumin, ethanol, and phosphatase inhibitor cocktail, LPS, penicillin and streptomycin were from Sigma. Blue RangeTM prestained protein molecular marker and BCA Protein Assay Kit were from Thermo Fisher Scientific. Anti-Akirin2 antibody (Cat. No. YN3819; 1:2000) was purchased from ImmunoWay Biotechnology Company (Newark, DE, USA). Anti-14-3-3θ antibody (Cat. No. ab14749; 1:1000), anti-IL-1β antibody (Cat. No. ab 200478; 1:1000), anti-NeuN antibody (Cat. No. ab177487; 1:200) and anti-β-catenin rabbit polyclonal antibody (Cat. No. ab16051, 1:1000) was purchased from Abcam (Cambridge, UK). Anti-IL-6 antibody (Cat. No. #12912; 1:400), anti-caspase 8 antibody (Cat. No. #8592; 1:800), anti-NF‐κB antibody (Cat. No. #6956; 1:800) and Anti-Rabbit IgG (H + L) F(abʹ)_2_ Fragment Alexa Fluor 647 conjugate (Cat. No. #4414; 1:800) were purchased from Cell Signaling Technology (Beverly, MA, USA). Anti-Iba1 antibody (Cat. No. 019-19741; 1:1000) was purchased from FUJIFILM Wako Pure Chemical Corporation (Osaka, Japan). Anti-PSD-95 antibody (Cat. No. 1:500) was purchased from Invitrogen (Eugene, Oregon, USA). HRP-conjugated Affinipure Goat Anti-Mouse IgG(H + L) (Cat. No. SA00001-1;1:3000), HRP-conjugated Affinipure Goat Anti-Rabbit IgG(H + L) (Cat. No. SA00001-2; 1:3000) were purchased from Proteintech (Chicago, IL). Alexa Fluor 647-conjugated Affinipure Goat Anti-Mouse IgG (H + L) (Cat. No. 115-605-003; 1:800) was purchased from Jackson ImmunoResearch Laboratories (West Grove, PA). PrimeSTAR DNA Polymerase, *SacI*,* XbaII*, *HindIII*, *BamHI* and T4 DNA ligase were purchased from Takara Bio, Inc. (Kusatsu, Japan). miR-203 mimics/inhibitors/scramble RNA were synthesized by GenePharma (Shanghai, China). The miR-203 mimics and the scramble RNAs were double-stranded (5ʹ-GUGAAAUGUUUAGGACCACUAG-3ʹ and 5ʹ-UUCUCCGAACGUGUCACGUTT-3ʹ, respectively). The miR-203 inhibitors were single-stranded RNA (5ʹ-CUAGUGGUCCUAAACAUUUCAC-3ʹ).

### Cell culture and transfection

The human embryonic kidney 293T cells, the murine macrophage cell line RAW264.7, and microglia cell line BV2 were purchased from ATCC and maintained in the lab. HEK 293T cells and RAW264.7 were grown in Dulbecco’s modified Eagles medium (DMEM) and the microglia cell line BV2 cells were grown in Dulbecco’s modified Eagles medium/nutrient mixture F-12 (DMEM/F12) supplemented with 10% fetal bovine serum, 100 U/mL penicillin and 100 µg/mL streptomycin (Biochrom KG, Berlin, Germany) at 37 °C in a humidified 5% CO_2_ incubator, respectively. Cells were transfected with the indicated constructs, microRNA mimics or scrambled miRNA using Lipofectamine™ 2000 transfection reagent (Invitrogen, Carlsbad, CA), according to the manufacturer’s instructions. RNA (pmol):lipofectamine (μL) of 20:1 and DNA (μg):lipofectamine (μL) of 1:2.5 were used in RNA and plasmid transfection, respectively. The cells were transfected for 24 h and 48 h before immunofluorescence and luciferase assay, respectively.

### RNA extraction and quantitative RT-PCR

Total RNA was extracted using TRIzol (Invitrogen) and 1 μg RNA was reversed transcribed using All-in-One™ miRNA First-Strand cDNA Synthesis Kit (GeneCopoeia, Rockville, MD) or PrimeScript RT reagent Kit with gDNA Eraser (Cat. No. RR047A, Takara, Kusatsu, Japan) according to the manufacturer’s instructions. Quantitative Real Time-Polymerase Chain Reactions (qRT-PCR) were performed using All-in-OneTM miRNA qPCR Kit (GeneCopoeia, Rockville, MD) for miR-203 on an Applied Biosystems Quanstudio3 (Applied Biosystems, Waltham, MA). The U6 small nuclear RNA was used as internal and the relative expression of miR-203 was calculated as described in our previous publication [17]. Quantitative Real Time-Polymerase Chain Reactions (qRT-PCR) were performed using TB Green Premix Ex TaqII (Tli RNaseH Plus) (Cat. No. RR820A, Takara, Kusatsu, Japan) for IL-1β with a primer pair of 5ʹ-GGGCCTCAAAGGAAAGAATCT-3ʹ and 5ʹ-GAGGTGCTGATGTACCAGTTGG-3ʹ. The quantification of IL-1β mRNA expression were calculated and normalized to the mRNA expression of glyceraldehyde-3-phosphate dehydrogenase (GAPDH), which was amplified with a primer pair of 5ʹ-TGAAGGGTGGAGCCAAAAG-3ʹ and 5ʹ-AGTCTTCTGGGTGGCAGTGAT-3ʹ.

### Dual-luciferase reporter gene assay

The target mRNA for the miR-203 and bind sites were predicted using the algorithms of TargetScan (http://www.Targetscan.org). *Akirin2* was recognized as the potential target of miR-203 with one conserved binding site at its 3ʹUTR. Primers targeting human *Akirin2* mRNA 3′UTR (forward primer: 5ʹ-CGAGCTCTACTGTTAGTGACTGATAAGGATGC-3ʹ; reverse primer, 5ʹ-GCTCTAGAACAAAGCATCTTAATGACACAGTA-3ʹ) and *14-3-3θ* mRNA 3′UTR (forward primer: 5ʹ-ATAAAGAGCTCTTCCTTCAAGAAACCTTTTTACACA-3ʹ; reverse primer: 5ʹ-ATAAATCTAGATGAAAGGAAACCCCCGAAG-3ʹ) were used to amplify the 227 bp segment of the *Akirin2* 3′UTR and 419 bp segment of the *14-3-3θ* 3ʹUTR containing the miR-203-binding sites from the cDNA library of 293T cells, respectively. These fragments were cloned into the SacI/XbaI sites of pmirGLO Dual-Luciferse miRNA target expression vector (Promega, Madison, WI). The primer pairs for Akirin2 3ʹUTR (forward primer: 5ʹ-ATAAAGGATCCGTGGGCTGCCTTGTTCCTTG-3ʹ; reverse primer: 5ʹ-ATAAAAAGCTTTAATGATACAGCAAAATGAGGCCAC-3ʹ) and for 14-3-3θ 3ʹUTR (forward primer: 5ʹ-ATAAAGGATCCTTCCTTCAAGAAACCTTTTTACACA-3ʹ; reverse primer: 5ʹ-ATAAAAAGCTTTGAAAGGAAACCCCCGAAG-3ʹ) were used to amplify 3ʹUTR of Akirin2 and 14-3-3θ containing miR-203 binding sites and were cloned into *HindIII*/*BamHI* sites in the pSliencer-4.1 vector (Thermo Fisher Scientific). All these primes and the relevant Mut-*Akirin2*-3ʹUTR and Mut-*14-3-3θ*-3ʹUTR with sense mutations at the seed sequences of the miRNA:mRNA interaction sites were synthesized by Sangon Biotech (Shanghai, China). All sequences were verified by sequencing (Sangon Biotech, Shanghai, China). 293T cells were co-transfected with miR-203 mimics or scrambled RNA with the indicated plasmids using Lipofectamine 2000 in 6-well petri dishes. Luciferase assays were performed 48 h post transfection using the Dual-Luciferase Reporter 1000 Assay System (Cat. No. #E2920, Promega). The luciferase activity was measured using the luminometer (CLARIOstarplus, BMG Labtech, Orthenberg, Germany) and were normalized to Renilla luciferase activity.

### Western Blot

Mouse hippocampus tissue and cells were lysed with RIPA buffer (including PMSF and phosphatase inhibitor cocktail). The protein concentration was detected using the BCA Protein Assay Kit (Thermo Fisher Scientific). Equal amount of proteins were separated on 10% sodium dodecyl sulfate-polyacrylamide gel and were transferred to PVDF membranes subsequently. The PVDF membranes were then blocking with 5% skim milk for 2 h under room temperature and were incubated with indicated primary antibodies at 4 °C overnight, followed by blotting with HRP-conjugated secondary antibody for 1 h at room temperature. The membranes were washed three times for 5 min each time with TBST before and after hybridization. The membranes were visualized by chemiluminescence (Immobilon horseradish peroxidase; Millipore, Billerica, MA) using Amersham Imager 680 (GE Healthcare Bio-Sciences Corp, Piscataway, NJ). The experiments were repeated for three times. Densitometry analysis was quantified using ImageJ v1.4.3.67 and expression of target proteins were normalized to the expression of β-actin.

### Immunofluorescence analysis of cultured cells

The cultured RAW264.7 and BV2 cells were transfected with indicated constructs. Cells were treated with or without 1 μg/mL lipopolysaccharide (LPS) for 30 min 24 h post transfection. Briefly washed with icy-cold phosphate buffer saline (PBS), cells were fixed in 4% paraformaldehyde for 15 min under room temperature and then permeabilized with 0.2% Triton X-100 for 15 min. The cells were then blocked in PBS containing 10% BSA and 22.52 mg/mL glycine and incubated with the appropriate dilutions of indicated primary antibodies at 37°C for 1 h. Excessive antibodies were washed off with PBS three times before incubation with an Alexa Fluor 647 (red)-conjugated secondary antibody at 37°C for 1 h. Cell nuclear was stained with Hoechst 33342 (1 μg/mL, Cat. No. B2261, Sigma-Aldrich) at room temperature for 10 min. Finally, the cells were visualized under appropriate wavelength on a laser confocal microscope (Olympus, FV3000, Tokyo, Japan). The images were captured using Olympus FV3000 confocal microscope and processed with FV31S-SW Viewer software (Ver.2.5). Five visions were randomly selected in each sample and experiments were repeated five times independently. The nuclear region of interest (ROI) was defined by Hoechst 33342 staining, which was identified using Image-Adjust-Color Threshold (ImageJ), followed by circumscribing the nuclear region automatically using Analyze–Analyze Particals (ImageJ). The fluorescence intensities of NF-κB within the nuclear region of interest were then calculated and analyzed by ImageJ software (National Institutes of Health, Bethesda, MD, USA).

### Construction of miR-203 lentiviral expression vector

The 76 bp mmu-pre-miR-203 (MI0000246) fragment with 100 bp flanking sequences at both 5ʹ and 3ʹ end was obtained by PCR amplification with primer pairs (forward primer: 5ʹ-GGAAAGGACGAAACACCGGCCGCACAGAGTGCAGCCCGGCC-3 ʹ; reverse primer, 5ʹ-TGTCTCGAGGTCGAAATTCAAAAAATCTCGGCGATCCGGGTGCCC-3ʹ) and cloned into *AgeI*/*EcoRI* restriction sites of lentiviral vector GV309-eGFP (Genechem, Shanghai, China). The sequences were verified by DNA sequencing. Viral packaging, purification, and tittering were performed as described previously [17]. Viral solutions with final titter of 4 × 10^8^ infectious units/mL were stored at – 80 °C before use.

### Stereotactic injection

The apparatus of stereotaxic surgery was setup as described previously [17]. In brief, the mice were anesthetized using isoflurane and head-fixed in the stereotact (Harvard Apparatus, Holliston, MA) over a heating pad set to 37 °C. The mice were injected with 1 μL viral solution each at both CA3 and dentate gyrus (DG) bilaterally at a rate of 0.5 μL/min using Hamilton 5 μL syringe (87930 Hamilton, Reno, NV) at the following coordinates: CA3 (− 2.0 mm at the anterior/posterior axis, ± 2.0 mm at the lateral/medial axis and − 2.0 mm at the dorsal/ventral axis relative to the bregma); DG (− 1.5 at the anterior/posterior axis, ± 1.5 at the lateral/medial axis and − 2.0 at the dorsal/ventral axis relative to the bregma in mm). The pipette was held in place for 5 min before retraction after each injection. Antibiotics (50 mg/mL, Amoxil) were administered for 7-day post-surgery. For intracerebral injection of LPS, mice were injected with 5 μg/2 μL of LPS at both sides of bilateral ventricle following coordinates: − 1.0 at the anterior/posterior axis, ± 0.3 at the lateral/medial axis and − 2.0 at the dorsal/ventral axis relative to the bregma in mm [18]. The mice were sacrificed, and the brain tissue was collected 24 h after the intracerebral injection of LPS.

### Brain tissue collection

Mice were anesthetized using isoflurane before transcardially perfused with 1 × PBS. Mouse brains were removed and the hippocampus were dissected. The hippocampus tissues were washed by PBS and diced into 15–20 pieces before homogenization in M tubes (130-093-236, Miltenyi Biotec) with 100 mg/1 mL ice cold lysis buffer (Cat. No. ab156034, Abcam, Cambridge, UK) containing 1:100 protease inhibitor cocktail (Cat. No. P2714, Sigma-Aldrich, St. Louis, MO). M tubes were then transferred onto gentleMACS Dissociator (130-095-937, Miltenyi Biotec) for homogenizing using the gentleMACS program Protein_01.01. The homogenate was transferred to 1.5 mL Eppendorf and centrifuged at 12,000*g* for 20 min. The supernatants were collected, aliquoted and frozen at − 80 °C before use.

### Immunohistochemistry

Mouse brains tissues were fixed in 4% paraformaldehyde overnight at 4 °C and then dehydrated with graded sucrose (10%, 20%, 30%, in sequence) in PBS at 4 °C. Brain tissues were the placed in O.C.T compound at − 20°C and stored at − 80°C. The brains were sectioned in the coronal plane at 15 µm thickness on a freezing microtome (CM1950, Leica), air dried, and washed with 1× PBS for 5 min. For immunostaining, the slides were permeabilized with 0.3% Triton X-100 for 15 min and blocked in PBS containing 1% BSA and 0.3% Triton X-100 for 2 h at room temperature. The slides were then incubated with primary antibody overnight at 4°C, followed by incubation with secondary antibody for 2 h at room temperature. Excess antibodies were washed off with PBS containing 0.3% Triton X-100 for three times at each interval. Cell nuclei were stained with Hoechst 33342 for 15 min under room temperature (1 μg/mL, Cat. No. B2261, Sigma-Aldrich). The slides were finally mounted in Fluoromunt™ Aqueous Mounting Medium (Cat. No. F4680; Sigma-Aldrich) and coverslipped before scanning with an Olympus FV3000 confocal microscope (Olympus Corporation, Tokyo, Japan). Bilateral hippocampal CA3 and DG fields of three 15 μm-thick consecutive coronal sections corresponding to bregma 1.82 mm to bregma 2.46 mm were captured at 20× magnification and digitized. The percentage of positively stained areas of Iba1, IL-1β, IL-6 or caspase 8 was quantified using ImageJ [19]. Three independent experiments were performed, and data was averaged per slide before statistical analysis.

### ImageJ skeleton analysis of microglia morphology

The brain tissue sections were prepared as described above and stained with anti-Iba1 monoclonal antibody for immunohistochemistry analysis. The images (15-μm z-stack at 0.8-μm intervals, 40×) were acquired with an Olympus FV3000 confocal microscope. ImageJ Skeleton analysis was performed according to the published protocols [20]. The images were converted to grayscale at first and the contrasts were adjusted to decrease the noise using the Unsharp Mask filter. Binary skeletonized images were then converted and the AnalyzeSkeleton plugin (http://imagejdocu.tudor.lu/) was subsequently applied to all skeletonized images to calculate the number of endpoints per frame, averaged over branch length. Three consecutive tissue sections from 1.82 to 2.46 mm rostral to bregma were chosen for each sample and the experiments were repeated for at least three times independently.

### TUNEL staining

Terminal-dexynucleotidyl transferase mediated nick end labeling (TUNEL) staining was utilized to evaluate the apoptotic cell death of mouse hippocampus tissue sections using In Situ Cell Death Detection Kit, TMR red (Roche, Cat. No. 12156792910). The slides were post-fixed with 4% paraformaldehyde for 20 min at room temperature and then washed for 30 min with PBS, followed by Proteinase K (1 μg/mL Cat. No. 1245680100, Merck, Darmstadt, Germany) treatment for 10 min at 37 °C. Briefly rinsed twice with PBS, the slides were incubated with 50 μL TUNEL reaction mixture for 1 h at 37 °C in a dark humidified chamber. The nuclei were stained with Hoechst 33342 and mounted with Fluoromunt™ Aqueous Mounting Medium (Sigma-Aldrich). The slides were visualized and analyzed using an Olympus FV3000 confocal microscope (Olympus Corporation, Tokyo, Japan). The images were captured using Olympus FV3000 confocal microscope and processed with FV31S-SW Viewer software (Ver.2.5). Bilateral hippocampal CA3 and DG fields of three 15 μm-thick consecutive coronal sections corresponding to bregma 1.82 mm to bregma 2.46 mm were captured at 20× magnification and digitized. Darkly and uniformly stained cells were counted as TUNEL-positive cells and were calculated manually using ImageJ [21]. The nuclei within the same view were counted automatically using ImageJ and the percentage of TUNEL-positive cells were calculated. Three independent experiments were performed for statistical analysis.

### Behavioral assessment

Mice were subjected to behavioral assessments 2-month post-stereotactic injection. The nesting behavior tests were performed as described previous studies [22]. Mice were transferred to individual testing cages equipped with a cotton nesting material (3 g/material) at 1 h before the dark phase. The intact cotton material was weighed, and the nest made from each mouse was scored 24 h later from 1 to 5. Briefly, the nestlet > 90% intact was scored 1, 50–90% intact scored 2, 10–50% intact scored 3. The nestlet < 10% intact and the torn material gathered within a quarter of the floor area of the cage, with < 50% of the nest circumference higher than mouse body, was scored 4. The nestlet < 10% intact and the formation of a crater with > 50% of the nest circumference higher than mouse body, was scored 5.

Fear conditioning test was performed 1 day after the nesting behavior test. In training phase, each mouse explored the experimental chamber (XRXC404, Xinruan Information Technology Co., Ltd., Shanghai, China) for 120 s before presentation of a 30 s auditory cue (2000 Hz, 85 dB). The tone was followed immediately by 2-s electrical foot shock (0.7 mA). The tone-foot shock pairings were repeated three times with I min intertrial intervals. The mice were returned to the home cages in 30 s after the training. The condition test was performed in 24 h after training, the mice were placed back to the chamber for 6 min without tone and foot shock and the times of freezing behaviors were recorded in an 2 s interval. The tone test was performed in 2 h after condition test, the mice were placed in a test chamber that was different from training and context test. 30 s auditory stimulus (2000 Hz, 85 dB) were given to the mice for three times with 1 min interval between each tone stimulation and the freezing behaviors were recorded for 270 s. Before each new training and testing, the chamber was cleaned with 70% ethanol to eliminate odors from the previous mouse. The freezing behaviors were recorded by SuperFcs software (Xinruan Information Technology Co., Ltd., Shanghai, China).

The short- and long-term spatial learning and memory were assessed using Barnes maze, which consists of a circular platform with one of the 20 equally spaced holes connected to a dark chamber, the target hole. Forced discovery of the target box was stimulated by light (200-W white-light lamp) and noise (85 dB). 2 min adaption was allowed for each mouse before the test. At the training sessions, each individual mouse went through a 4-day spatial acquisition phase, during which each mouse had a 3 min trial twice per day with 15 min interval between each trail. Each individual mouse was placed in the center of the platform and covered with a white chamber for 5 s. The test started by removing the chamber, and the mouse were allowed for 3 min free exploring. The aversive noise and light were subsequently switched on to encourage the mouse to run and escape into the target box. The mouse was gently guided to the target hole by the examiner when failed to enter the target hole within 3 min. The mouse was then returned to its home cage and the platform was cleaned with 70% ethanol. On the completion of the 4-day reference memory phase, the mice were tested on day 5 for the short-term retention and on day 12 for the long-term retention. The latency to find the target hole in each trial were recorded using ANY-maze video tracking system (Stoelting Co., USA). All the behavioral assessments were evaluated by an observer who was blind to the animal’s information.

### Statistical analysis

All data presented as mean ± Standard deviation (S.D.). Statistical analysis was performed using GraphPad Prism 6.0 software (GraphPad, San Diego, CA, USA). Comparisons between two groups were analyzed using Student’s *t* tests (unpaired, 2-tailed). For multiple comparisons, one-way ANOVAs followed by post hoc test were used. The statistical chart was made by GraphPad Prism 6.0 software. Differences were considered statistically significant at *P* < 0.05.

## Results

### Akirin2 was recognized as a new target of miR-203

**Fig. 1:**
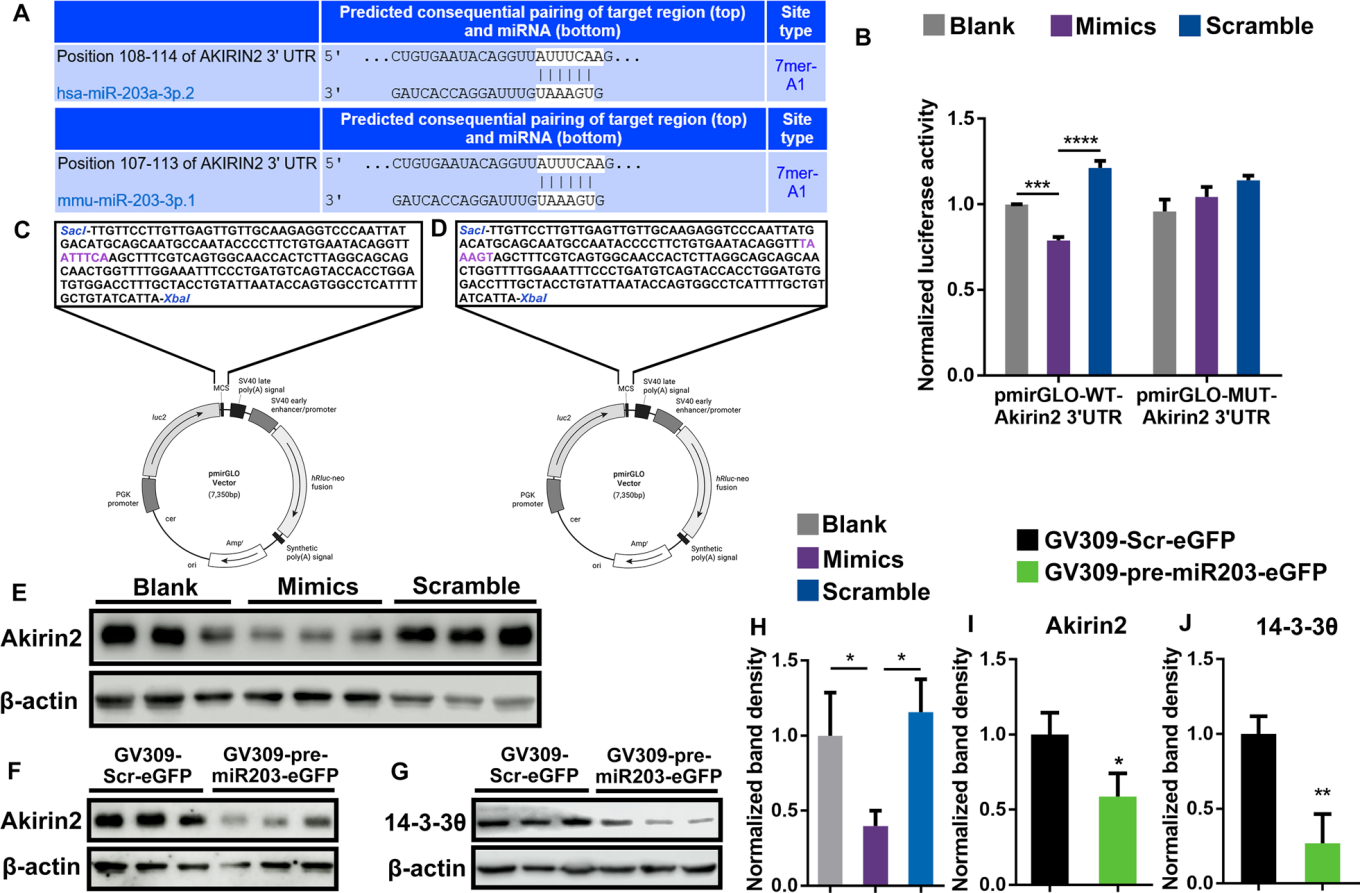
miRNA-203 targeted downregulation of Akirin2 expression in vivo and vitro. **A** The conserved miRNA:mRNA interaction site in the 3ʹUTR of Akirin2 predicted using TargetScan. **B** Luciferase assay showed a direct sequence-specific targeting of miR-203 on Akirin2 3ʹUTR. The results were presented as means of normalized luciferase activities of three biological replicated each measured in triplicates, with error bars showing the standard deviation. Asterisks indicate statistical significance (ANOVA, Turkey’s post hoc). **C** and **D** The 217 bp Akirin2 3ʹUTR containing human wild-type (**C**) or sense mutation in the seed sequence of the miR-203 binding site (**D**) were cloned into pmiRGLO and co-transfected into 293T cells with miRNA-203 mimics or nonsense scramble RNA, respectively. Luciferase activities were measured 48 h post transfection. **E** and **H** Downregulated Akirin2 expression was detected 48 h post-transfection using western blots in cultured BV2 cells transfected with miRNA-203 mimics or scramble RNA, respectively. β-Actin was used as an internal control. Luminescence-based relative quantification of protein (Akirin2:β-actin) of three individual biological replicates with error bars representing the standard deviation showed significant decreasing of Akirin2 expression in cells transfected with miR-203 mimics (ANOVA, Turkey’s post hoc). **F** and **I** Intrinsic expression of Akrin2 was decreased in mouse hippocampus with stereotactic injection of GV309-pre-miR203-eGFP, comparing with that in control mice injected with GV309-Scr-eGFP. Results were presented as the luminescence-based relative quantification of western blots of protein (Akirin2:β-actin) of three individual biological replicates with error bars showing the standard deviation. **G** and **J** Intrinsic expression of 14-3-3θ was decreased in mouse hippocampus with stereotactic injection of GV309-pre-miR203-eGFP, comparing with that in control mice injected with GV309-Scr-eGFP. Results were presented as the luminescence-based relative quantification of western blots of protein (14-3-3θ:β-actin) of three individual biological replicates with error bars showing the standard deviation. Asterisks indicate statistical significance between samples (*P < 0.05; **P < 0.01; ***P < 0.001; ****P < 0.0001)

In silico analysis using Targetscan predicted Akirin2 to be a potential target of human miR-203 and identified one putative miRNA:mRNA interacting site within its 3ʹUTR, possessing a 7-mer seed sequence that was conserved between human and mouse (Fig. 1A).

227 bp Akirin2 3ʹUTR containing either wild type seed sequence or sense mutation of the seed sequence was cloned into the SacI/XbaI sites of pmirGLO to generate pmirGLO-WT-Akirin2 3ʹUTR and pmirGLO-MUT-Akirin2 3ʹUTR, respectively (Fig. 1C, D). Co-transfection of miR-203 mimic and pmirGLO-WT-Akirin2 3ʹUTR resulted in significant down-regulation of luciferase activity in 293T cells 48 h post transfection, comparing with the blank controls and scramble transfections, respectively. Transfection of pmirGLO-MUT-Akirin2 3ʹUTR resulted in no difference in luciferase activity between miR-203 and scramble miRNA transfections (Fig. 1B). miR-203 mediated expression repression of Akirin2 was further verified both in vitro and in vivo using western Blots. The Akirin2 proteins were significantly reduced in cultured BV2 cells transfected with miR-203 mimics (*P* < 0.05), comparing with that in nonsense transfected controls and in blank controls (Fig. 1E, H). The hippocampus of mice with stereotactic injection of lentiviral vectors expressing pre-miR203 or scrambled control sequences were collected 2-month post-infection (Additional file 1: Fig. S1A). Ectopic overexpression miR-203 in the hippocampus infected with GV309-pre-miR-203-eGFP was confirmed by quantitative RT-PCR (Additional file 1: Fig S1B). In consistence with the results of in vitro assay, an averaged 40% reduction (*P* < 0.05) of Akirin2 protein levels was observed in the hippocampus overexpressing miR-203, comparing with that in the hippocampus infected with control lentivirus (Fig. 1F, I). The repressed protein expression of 14-3-3θ was again demonstrated in the hippocampus overexpressing miR-203, in consistent with the result in our previous publication (Fig. 1G, J).

### miRNA-203 promote NF‐κB signaling via Akirin2-independent pathway

**Fig. 2:**
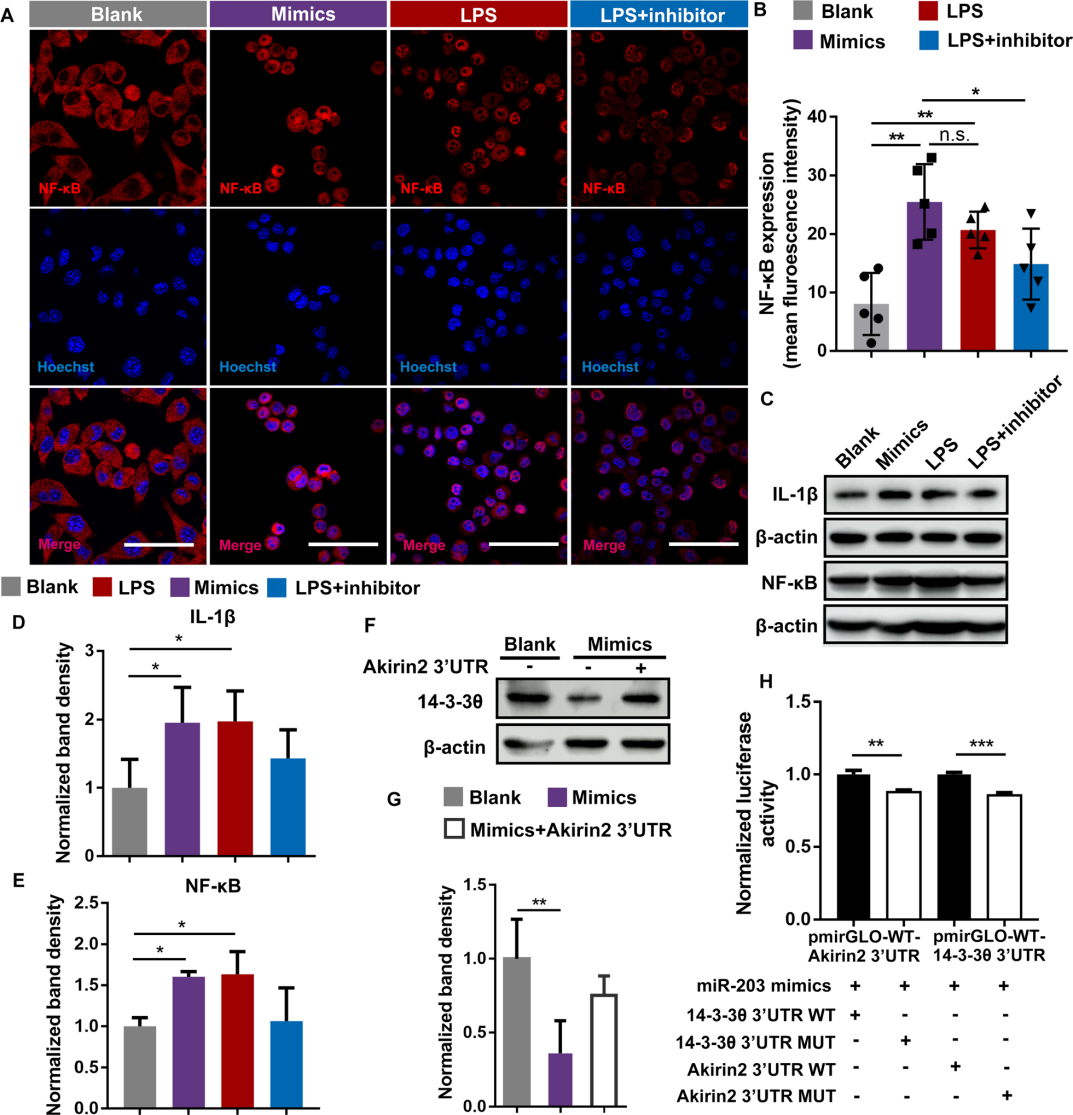
Overexpression of miRNA-203 promote the nuclear translocation of NF‐κB. **A** The cultured BV2 cells were transfected with miRNA-203 mimics or miRNA-203 inhibitors. Mock transfections were performed in the blank controls and LPS-induced NF‐κB translocation was used as the positive control. Subcellular localization of endogenous NF-κB was visualized by immunofluorescent using monoclonal antibody of the p65 subunit of NF‐κB and a Alexa Fluor 647-labeled secondary antibody (red). **B** The fluorescent intensities of NF‐κB in the nuclei, defined by Hoechst 33342 staining (blue), were quantified and the results of five independent experiments were presented, with the height of columns representing the mean and the error bars showing the S.D. The statistical significance between samples were indicated by asterisks (ANOVA, Turkey’s post hoc). The scale bars were 50 μm. **C** Western blots showed increased expression of IL-1 β and NF-κB in BV2 cells transfected with miR-203 mimics, as well as in LPS-induced cells. Untransfected cells were used as the negative controls. Transfection of miR-203 inhibitors reversed the LPS-induced elevation of IL-1β and NF-κB. **D** and **E** Luminescence-based relative quantification of protein (IL-1 β:β-actin; NF-κB:β-actin) were expressed as mean ± S.D. of three biological replicates (ANOVA, Dunnett’s post hoc). **F** HEK293T cells were transfected with miRNA-203 mimic with or without and pmirGLO vector containing wild type Akirin2 3ʹUTR. The intrinsic levels of 14-3-3θ were detected using western blots. Mock transfection was performed in blank controls. **G** Luminescence-based relative quantification of protein (14-3-3θ: β-actin) was performed in three individual biological replicates, with error bars representing the standard deviation (ANOVA, Turkey’s post hoc). Downregulated expression of 14-3-3θ in cells transfected with miR-203 mimics was reversed by overexpression of Akirin2 3′UTR. H Akirin2 3ʹUTR and 14-3-3θ 3ʹUTR Luciferase activity in 293T cells transfected with miR-203 mimics and pSilencer 4.1 containing wild type or mutant form of Akirin2 3ʹUTR and 14-3-3θ 3ʹUTR. Asterisks indicate statistical significance between samples (*P < 0.05; **P < 0.01; ***P < 0.001)

Given the miR-203 mediated down regulation of Akirin2 observed both in vitro and in vivo, we initially proposed a negative role of miR-203 in regulating NF‐κB signaling. To our surprise, analysis of the immunofluorescence intensities revealed that transfection of miR-203 promoted the nuclear translocation of NF‐κB in cultured BV2 and RAW264.7 cells, while transfection of miR-203 inhibitor repressed the LPS induced NF‐κB translocation (Fig. 2A, B, Additional file 2: Fig. S2A, B). Expression of NF-κB and the pro-inflammatory cytokine IL-1β, which produced upon NF‐κB activation, was further evaluated using western blot in both BV2 cells and RAW264.7 cells that transfected with miR-203 mimics or LPS-induced cells transfected with miR-203 inhibitors (Fig. 2C, Additional file 2: Fig. S2C). The LPS induction was used Quantification of the relative protein expressions revealed significant elevation of NF-κB and IL-1β expression in cells transfected with miR-203 mimics, as well as in LPS-induced cells (Fig. 2D, E, Additional file 1: Fig. S2D). Transfection of miR-203 inhibitors reversed the LPS-induced elevation of NF-κB and IL-1β, although no statistical significance was shown yet (Fig. 2D).

Given the positive role Akirin2 played in NF‐κB signaling, miR-203 was likely to activate NF‐κB signaling via an Akirin2-independent pathway. This hypothesis was evident by the significant downregulation of another protein target of miR-203, 14-3-3θ, which was a negative regulator of NF-κB, in cells transfected with miR-203 mimics (Fig. 2F, G). Overexpression of Akirin2 3ʹUTR restored the down-regulated 14-3-3θ protein expression in cells transfected with miR-203 mimics, suggesting a competition for miR-203 binding between these two protein targets (Fig. 2F, G). The potential competition for miR-203 interaction between these targets were further verified using luciferase assay. Mutual competition for miR-203 targeting was displayed between the 3ʹUTR of 14-3-3θ and Akirin2, while the 3ʹUTR containing mutation in the seed sequences for miR-203 targeting displayed attenuated binding for miR-203 (Fig. 2H).

14-3-3 is abundant in brain and interact with numerous proteins that have various biological functions, while in most cases are ascribed to neuroprotective functions [23]. 14-3-3θ was a definite target gene for miRNA-203 and displayed inhibitory effect on TLR2-induced NF-κB activation [24]. The overall pro-inflammatory role of miR-203 was likely to be Akirin2-independent and could at least partly be explained by the relative abundance of these proteins and their potential competition for miR-203 targeting.

### Overexpression of miR-203 induced microglial activation and the production of pro-inflammatory cytokines in mouse hippocampus

**Fig. 3:**
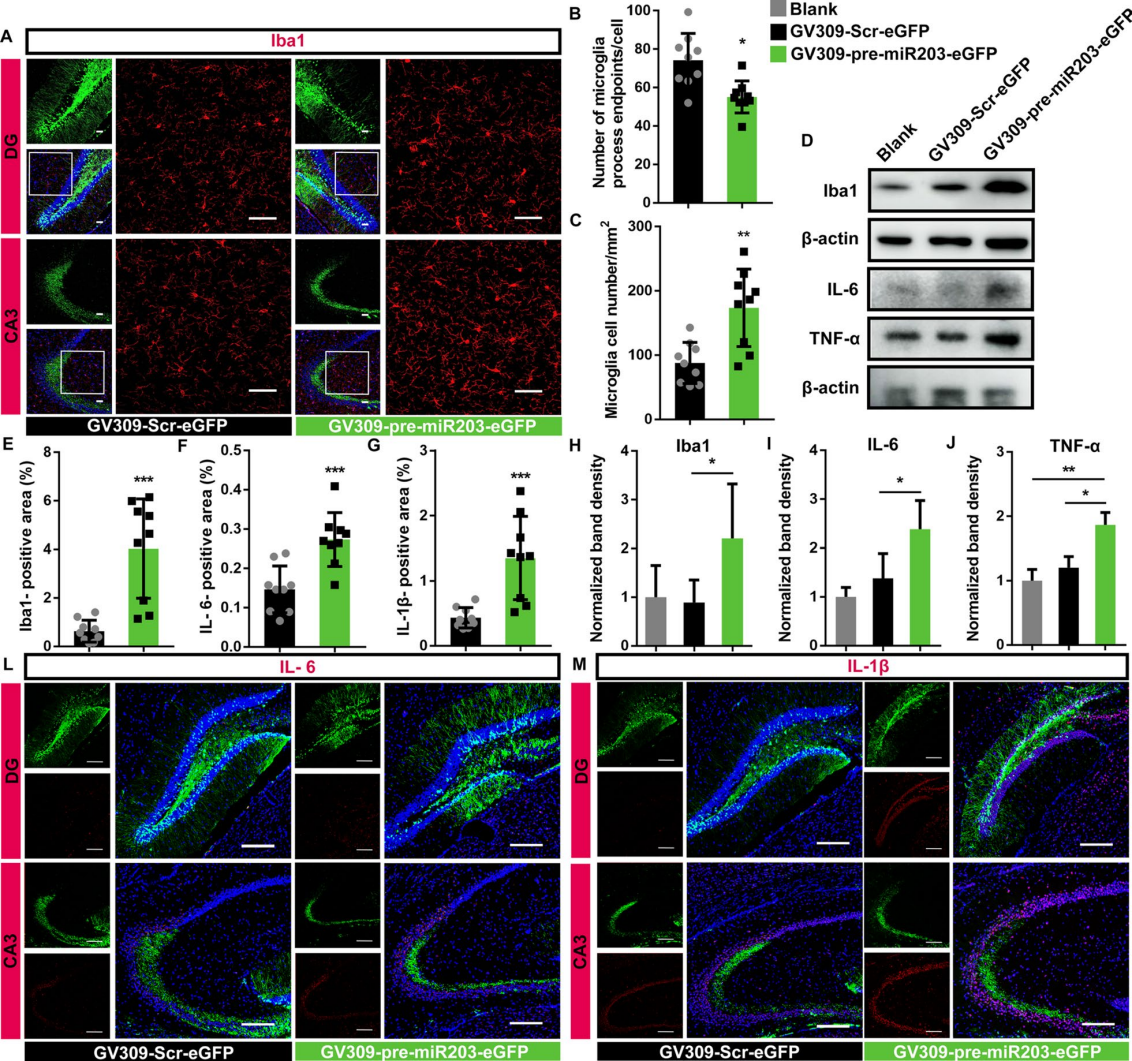
miRNA-203 activated microglia and promoted inflammatory gene expression. **A** Representative confocal Z-stack images for Iba1 (red) in CA3 and DG of mouse hippocampus overexpressing miR203 or nonsense scrambles. The scale bar was 50 μm. **B** The skeleton analysis of microglia morphology. Summed microglia endpoints were reduced in the mouse hippocampus with ectopic expression of miR-203, compared with controls expressing nonsense scrambles. Results of three consecutive sections consisting of four views of bilateral CA3 and DG of three independent biological replicates were presented, with the height of columns representing the mean and the error bars showing the S.D. **C** Quantification of microglia cell numbers. Results of three consecutive sections consisting of four views of bilateral CA3 and DG of three independent biological replicates were presented, with the height of columns representing the mean and the error bars showing the S.D. **D**, **H**–**J** Western blots showed increased expression of Iba1, IL-6 and TNF-α in mouse hippocampus with ectopic expression of miR-203, compared with that of controls expressing nonsense scrambles and untransfected blank controls. Luminescence-based relative quantification of protein (Iba1:β-actin; IL-6:β-actin; TNF-α:β-actin) are expressed as mean ± SD of three biological replicates (**H**–**J**) (one-way ANOVA, Turkey’s post hoc). **E** Area percentages of Iba1 (red) in the DG and CA3 were significantly increased in mouse hippocampus with ectopic expression of miR-203, compared with that of controls expressing nonsense scrambles. Results of three consecutive sections consisting of four views of bilateral CA3 and DG of three independent biological replicates were presented, with the height of columns representing the mean and the error bars showing the S.D. **F**, **L** Immunofluorescence showed increased IL-6 (red) in mouse hippocampus with ectopic expression of miR-203 (green), compared with controls expressing nonsense scrambles. Quantification of IL-6 positive areas of three consecutive sections consisting of four views of bilateral CA3 and DG of three independent biological replicates were presented, with the height of columns representing the mean and the error bars showing the S.D. **G**, **M** Immunofluorescence showed increased IL-1β (red) in mouse hippocampus with ectopic expression of miR-203 (green), compared with controls expressing nonsense scrambles. Quantification of IL-1β positive areas of three consecutive sections consisting of four views of bilateral CA3 and DG of three independent biological replicates were presented, with the height of columns representing the mean and the error bars showing the S.D. Nuclei were stained with Hoechst 33342 (blue) and the scale bar was 150 μm. Asterisks indicate statistical significance between samples (*P < 0.05; **P < 0.01; ***P < 0.001)

Lentiviral expression vector GV309-pre-miR-203-eGFP was introduced to the CA3 and DG sub region of mouse hippocampus on both sides using stereotactic injections. Immunohistochemistry analysis was performed with anti-Iba1, followed by staining with the Alexa Fluor 647-conjugated Affinipure Goat Anti-Mouse IgG (H + L) (Cat. No. 115-605-003; 1:800) on the frozen sections of hippocampus (Fig. 3A). Microglial morphology evaluation using ImageJ Skeleton analysis displayed increased ramification and endpoints in mouse hippocampus overexpressing miR-203, comparing with the controls overexpressing scramble miRNA (Fig. 3B, Additional file 3: Fig. S3). Quantification of microglia revealed a significant increase in microglia cell number and in the fluorescence intensity of Iba1 in the CA3 and DG regions of mouse hippocampus overexpressing miR-203 (Fig. 3C, E). Activation of microglia was also verified by the significantly elevated protein level of Iba1 in mouse hippocampus overexpressing miR-203, shown by western blot assay (Fig. 3D, H). Activation of microglia induced by miR-203 resulted in elevated inflammatory response in the relevant regions in mouse hippocampus, evidenced by immunohistofluorescence analysis using anti-IL-6, anti-IL-1β, and fluorescent secondary antibody Alexa Fluor 647-conjugated Affinipure Goat Anti-Mouse IgG (H + L) (Cat. No. 115-605-003; 1:800). The fluorescence intensities of both proinflammatory cytokines were significantly increased in the regions in mouse hippocampus infected with GV-309-pre-miR-203-eGFP (Fig. 3F, G, L, M). The elevated expression of pro-inflammatory cytokines (IL-1β and TNFα) in mouse hippocampus overexpressing miR-203 were also verified using western blot (Fig. 3D, I, J). We also investigated the miR-203 expression under brain insult in the LPS-induced neuroinflammation using quantitative real-time PCR. Dramatically elevated miR-203 expression was observed in the mouse brain induced by LPS (Additional file 1: Fig. S1C, D).

### Overexpression of miR-203 resulted in neuronal cell death and impairment of neuronal function

**Fig. 4:**
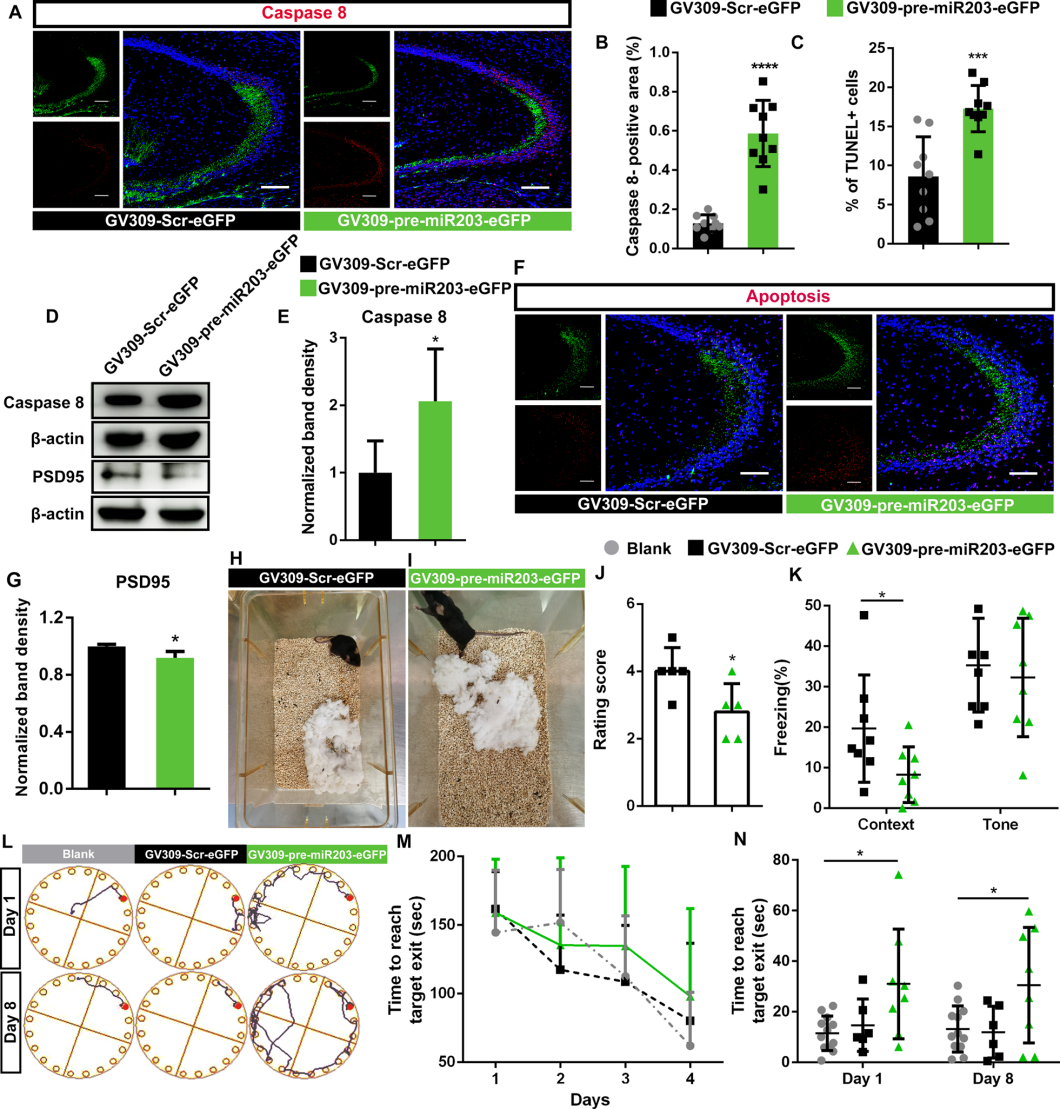
Overexpression of miRNA-203 induced neuronal cell death and caused cognitive impairment. **A** and **B** Representative immunofluorescence images showing ectopic expression of miR-203 (green) induced elevation of caspase 8 (red) in the hippocampal CA3. The expression of Caspase 8 was primarily distributed around miR-203 overexpressing cells. Quantification of Caspase 8 positive areas of three consecutive sections consisting of four views of bilateral CA3 and DG of three independent biological replicates were presented, with the height of columns representing the mean and the error bars showing the S.D. **C** and **F** Immunofluorescent images of TUNEL assay representing the increased apoptosis in the CA3 regions of mouse hippocampus with ectopic expression of miR-203 (green). Quantification of apoptotic cells (red) of three consecutive sections consisting of views of bilateral CA3 of three independent biological replicates were presented, with the height of columns representing the mean and the error bars showing the S.D. **D**, **E**, **G** Western blots showing the upregulated caspase 8 and downregulated PSD95 in mouse hippocampus with ectopic expression of miR-203, compared with that of mouse hippocampus expressing nonsense scrambles. Luminescence-based relative quantification of proteins was expressed as mean ± S.D. of three biological replicates. **H**, **I**, **J** Decreased nest building qualities were observed in mice with hippocampal overexpression of miR-203 (**I**), compared with controls overexpressing nonsense scrambles (**H**). Results of scoring of a 5-point rating scale were expressed as mean ± S.D. of five biological replicates, with the score of nest quality of each mouse indicated (**J**). **K** Fear conditioning test showed decreased freezing behavior in mice with hippocampal overexpression of miR-203 in the context-related test, compared with controls overexpressing nonsense scrambles. No difference in the freezing behavior in the tone-related fear conditioning test was observed (*n* = 8). **L** The representative tracks of a blank control mouse, a mouse overexpressing scramble microRNA and a mouse overexpressing miR-203 in the hippocampus on day 1 and day 8 of the probe trails in Barnes maze. **M** Latency to identify the target holes in the training sessions of Barnes maze. Results were presented as the group mean ± S.D. **N** The time spent to find the target holes. The results of each tested mouse were present, with the center line represents the mean, and error bars show mean ± S.D. (one-way ANOVA, Turkey’s post hoc). Nuclei were stained with Hoechst 33342 (blue) and the scale bar was 150 μm. Asterisks indicate statistical significance between samples (*P < 0.05; ****P < 0.0001)

Given the miR-203 induced elevation in inflammatory response observed in the hippocampus, we further investigated the putative impact of miR-203 over expression on neuron function. Post Synaptic Density 95 protein (PSD-95) is an important scaffolding protein enriched in the post postsynaptic density in the excitatory synapses in hippocampus circuits, while disruption of PSD95 associates with cognitive dysfunction and learning deficits [25]. Immunohistochemistry analysis of the frozen section of mouse hippocampus using anti-Caspase8 and fluorescence secondary antibody Alexa Fluor 647-conjugated Affinipure Goat Anti-Mouse IgG (H + L) (Cat. No. 115-605-003; 1:800) demonstrated significantly increased Caspase 8 expression in the CA3 and DG sub region induced by miR-203 overexpression (Fig. 4A, B, Additional file 4: Fig. S4A, B). Comparing with that of the mouse hippocampus overexpressing nonsense control miRNA, upregulation of Caspase8 expression and downregulation of PSD95 expression in the tissue of mouse hippocampus infected with GV309-pre-miR203-eGFP were further shown using western blot (Fig. 4D, E and G). Whether miR-203 induced elevation of neuroinflammation eventually resulted in neuronal cell death was evaluated using TUNEL assay. Our results showed significantly increased apoptosis accompanied with the ectopic overexpression of miR-203 (Fig. 4C, F, Additional file 4: Fig. S4C, D).

To verify the influences of miR-203 overexpression on hippocampal neuron activities, mouse behavioral assessments were performed 2 months after stereotactic injection. In the nesting behavioral test, significant differences in nest building quality between the GV309-pre-miR203-eGFP and GV309-Scr-eGFP groups were identified in the examination of nest building behavior. The GV309-Scr-eGFP group had better performance in nest-building than that of the GV309-pre-miR203-eGFP mice (Fig. 4H–J). In addition, test in fear conditioning showed the GV309-pre-miR203-eGFP group had less freezing behavior in the context-related test compare with the GV309-Scr-eGFP controls, while no difference in the freezing behavior in the tone-related fear conditioning test was observed between the mice overexpressing miR-203 and the control mice (Fig. 4K). The contextual fear is thought to only involve the hippocampus, whereas cued fear is more reliant on the amygdala [26]. Impaired spatial learning and memory for mice overexpressing miR-203 were demonstrated using Barnes maze. Significantly increased latency to identify the target holes was observed on the probe trails on day 1 and day 8, reflecting the short- and long-term memory, respectively (Fig. 4L–N). Our results implicated the impaired neuron activities and cognitive dysfunction of mice with ectopic miR-203 expression in the hippocampus.

## Discussion

There are numerous studies on the biological function of miR-203, which has a profound function in regulating cell proliferation and inflammation in both physiological conditions as well as in pathological progression of various diseases. Expression of miR-203 was enriched in keratinocytes and upregulated in inflammatory skin conditions, such as psoriatic lesions, where the expression of a series of cytokines was differentially regulated by miR-203 [14]. Aberrant expression of miR-203 was also reported in the tumorigenesis and cancer progressions of various tissue origins [27–30]. Contradictory results were presented in these studies with respect to the exact biological functions of miR-203 in regulating cell proliferation and cell inflammatory responses, at least partly explained by multiple downstream targets that had opposite impact on overlapping pathways. For instance, both miR-203 induced promotion and inhibition of cell proliferation were observed, in hepatocytes and pancreatic cancer cells, respectively, via targeting PTEN and SLUG [31, 32]. Similarly, both pro-and anti-inflammatory functions were ascribed to miR-203 via modulating negative and positive regulators of cytokine signaling [33–35].

The importance of the biological role of miR-203 in CNS has been noticed and extensively investigated recently. Elevated expression of miR-203 was first reported in isoflurane induced B35 cells as well as in the cerebral cortex of rats in our previous study [36]. Subsequent studies on the cellular functions of miR-203 suggested both neuroprotective and neurodisruptive effects via targeting different signaling molecules, such as c-Jun, neuronal growth regulator 1 (NEGR1), bcl-2, as well as 14-3-3 that verified in our recent research [17, 37–39].

In this study, we identified a novel direct target of miR-203 in CNS, Akirin2, which interact with the p50 subunit of NF‐κB and direct its translocation to the nucleus as a complex of NF‐κB-Akrin2-SWI/SNF to activate pro-inflammatory gene expression [16]. However, overexpression of miR-203 promoted NF‐κB signaling via Akirin2-independent pathway in vitro and displayed pro-inflammatory effects in vivo in mice with ectopic miR-203 expression in hippocampus. Given that multiple factors in NF‐κB-dependent inflammatory pathways, such as IL-24, IL-8, MyD88, and SOCS family etc., were recognized as direct targets of miR-203, we proposed that the involvement of other protein factors substantially contribute to outcomes of the influence of miR-203 in regulating inflammatory response. 14-3-3 protein family is highly abundant in CNS and conserved in all eukaryotes. These proteins function as scaffolding proteins and interact with a wide range of serine/threonine phosphatases and kinases, as well as act as molecular chaperones to prevent the aggregation of misfolded proteins [40]. Although the detailed functions of 14-3-3 were behind the mist, neuroprotective roles were ascribed to 14-3-3 in various pathological conditions, for instance, prevent phosphorylated tau aggregation in neurofibrillary tangles in Alzheimer disease and prevent α-synuclein aggregation in Parkonson’s disease [23]. Isoforms of 14-3-3 protein were also implicated in repressing NF-κB activity by tethering p65 subunit in cytoplasm [41]. In addition, 14-3-3θ was shown to negatively regulated TLR2-dependent NF-κB signaling and was identified as a direct target of miR-203 in CNS in vivo in our recent publication [17, 24]. Hence, the putative competition for miR-203 bind between 14-3-3θ and Akirin2 was investigated in this study and our results show the reversal of down-regulated 14-3-3θ protein in cells transfected with miR-203 mimics by overexpressing Akirin2 3ʹUTR. Taken together with the recent findings of other laboratories that indicating miR-203 as both suppressor and activator of neuroinflammation via targeting MyD88 and MEF2C, respectively, data in this research suggested that miR-203 is a key player in regulating inflammatory response as well as maintaining the immune homeostasis of CNS [35]. miR-203 possesses both anti- and pro-inflammatory properties and our identification of Akirin2 as a novel target of miR-203 adds complexity in this complex regulatory network of immune response. In BV2 cells and in CNS, enriched negative regulators such as 14-3-3θ might be the main target of miR-203 and compete for its targeting. Taken together the dramatically downregulated expression of 14-3-3θ in both BV2 cells and mouse hippocampus overexpressing miR-203, this could at least partly explain the overall pro-inflammatory role of miR-203 observed in vitro and in vivo.

Elevated neuron cell death and impaired neuronal activity in mouse model overexpressing miR-203 in the hippocampus were further displayed by TUNEL assay and PSD95 immunoblot, in agreement with the results from Swarup et al., who suggested that miR-203 was a hub regulatory miRNA in regulating apoptosis and was associated with the disease progression of frontotemporal dementia [13]. We further demonstrated that the mice overexpressing miR-203 in the hippocampus had impaired cognitive function, which was not addressed by Swarup et al. [13] in their mouse model with overexpressed miR-203 in frontal cortex. The involvement of miR-203 in regulating the inflammatory response in CNS was further evidenced by the notion of the significantly upregulated miR-203 expression in the nonneuronal cells of LPS-induced murine neuroinflammation. By juggling various components in NF-κB signaling, we suggested that miR-203 was also likely to be a key regulator to fine adjust neuroinflammation, which could be both the cause and consequence of apoptosis, and vice versa. The precise function of miR-203 should be addressed in specified context and attempts to use this microRNA as a potential therapeutic target should be planned with extra caution.

## Conclusion

Akirin2, a nuclear protein bridging NF-κB and chromatin remodeling complexes to regulate inflammatory gene expression, was identified as a novel target of miR-203 in CNS. Competition for miR-203 binding was displayed between Akirin2 3ʹUTR and 14-3-3θ, which was a negative regulator of NF-κB signaling. miR-203 displayed an overall pro-inflammatory role and increased apoptosis in the hippocampus of mice with ectopic overexpression of miR-203. Impaired neuronal activities and cognitive dysfunction were revealed by behavior assessment using nesting behavior tests and fear conditioning test. Together with the previous finding of other laboratories, our results support miR-203 as a key modulator in fine-tunning neuroinflammation via targeting different components in NF-κB signaling.

## Supplementary Information

**Additional file 1: Figure S1.** Stereotactic injection of lentiviral expression vector of GV-309-pre-miR-203-eGFP in the CA3 and DG sub regions of mouse hippocampus. A Bilateral stereotactic injection of GV309-pre-miR-203-eGFP into CA3 (− 2.0 mm at the anterior/posterior axis, ± 2.0 mm at the lateral/medial axis and − 2.0 mm at the dorsal/ventral axis relative to the bregma) and DG (− 1.5 at the anterior/posterior axis, ± 1.5 at the lateral/medial axis and − 2.0 at the dorsal/ventral axis relative to the bregma in mm) in the hippocampus. The scale bar was 200 μm. B Real-time quantitative PCR analysis showing the overexpression of miR-203 in the mouse hippocampus infected with GV309-pre-miR-203-eGFP, compared with controls infected with GV309-scr-eGFP. (*n* = 3). C Immunofluorescence images showing NeuN positive cells (red) in mouse hippocampus with stereotactic injection of GV309-Scr-eGFP or GV309-pre-miR203-eGFP (green). The lentiviral vector nonselective infected both neuron and nonneuronal cells. The scale bar was 200 μm. D and E Real-time quantitative PCR analysis showing the enrichment of miR-203 expression was significantly elevated in the LPS insulted mouse brains, comparing with the control. (*n* = 3). Asterisks indicate statistical significance between samples (*, *P* < 0.05; ***, *P* < 0.0001).

**Additional file 2: Figure S2.** Overexpression of miRNA-203 promote the nuclear translocation of NF‐κB in RAW 264.7. A The cultured RAW 264.7 cells were transfected with miRNA-203 mimics or miRNA-203 inhibitors. Mock transfections were performed in the blank controls and LPS-induced NF‐κB translocation was used as the positive control. Subcellular localization of endogenous NF‐κB was visualized by immunofluorescent using monoclonal antibody of the p65 subunit of NF‐κB and a Alexa Fluor 647-labeled secondary antibody (red). B The fluorescent intensities of NF‐κB in the nuclei, defined by Hoechst 33342 staining (blue), were quantified and the results of five independent experiments were presented, with the height of columns representing the mean and the error bars showing the S.D. The statistical significance between samples were indicated by asterisks.(one-way ANOVA, Turkey’s post hoc). The scale bars were 150 μm. C Western blots analysis of the protein expression of NF‐κB and IL-1β in RAW264.7 cells transfected with miR-203 mimics, in RAW264.7 cells treated with LPS and in LPS-induced RAW264.7 cells transfected with miR-203 inhibitor. Mock transfection was used as blank control and the expression of β-actin was used as internal control of protein expression. D and E Luminescence-based relative quantifications of protein were expressed as mean ± SD of three biological replicates (one-way ANOVA, Dunnett’s post hoc). Comparing with those in control cells, transfection of miR-203 mimics upregulated the expression of NF‐κB and IL-1β. LPS-induced cells were used as a positive control. Asterisks indicate statistical significance between samples (*, *P* < 0.05, **, *P* < 0.01; ****, *P* < 0.0001)

**Additional file 3: Figure S3.** 3D reconstruction of the z-stack images of immunofluorescence using anti-Iba1, showing the activation of microglia from mouse hippocampus overexpressing miR-203 (A) or overexpressing scramble RNAs (B).

**Additional file 4: Figure S4.** Overexpression of miRNA-203 induced neuronal cell death in DG regions of mouse hippocampus. A Representative immunofluorescence images showing miR-203 (green) induced elevation of caspase 8 (red) in the hippocampal DG. B Quantification of Caspase 8 positive areas of three consecutive sections consisting of views of bilateral DG of three independent biological replicates were presented as mean ± SD. C Immunofluorescent images of TUNEL assay representing the increased apoptosis in the DG regions of mouse hippocampus with ectopic expression of miR-203. D Quantification of TUNEL positive cells of three consecutive sections consisting of views of bilateral DG of three independent biological replicates were presented as mean ± SD. Asterisks indicate statistical significance between samples (***, *P* < 0.001)

## Data Availability

The data sets used and/or analyzed during the current study are available from the corresponding author on reasonable request.

## Abbreviations

CNS: Central nervous system
ROS: Reactive oxygen species
BBB: Blood–brain barrier
AD: Alzheimer’s disease
PD: Pakinson’s disease
M2: Microglia cells
PAMPs: Pathogen-associated molecular patterns
DAMPs: Damage-associated molecular patterns
PRRs: Pattern recognition receptors
TLR: Toll-like receptors
IKK: Inhibitor of κB kinase
FTD: Frontotemporal dementia
NIH: National Institutes of Health
FBS: Fetal bovine serum
DAB: 3,3-Diaminobenzidine tetrachloride
DTT: 1,4-Dithiothreitol
DMSO: Dimethyl sulfoxide
DMEM: Dulbecco’s modified Eagles medium
DMEM/F12: Dulbecco’s modified Eagles medium/nutrient mixture F-12
LPS: Lipopolysaccharide
PBS: Phosphate buffer saline
ROI: Region of interest
DG: Dentate gyrus
TUNEL: Terminal-deoxynucleotidyl transferase mediated nick end labeling
PSD-95: Post Synaptic Density 95 protein
NEGR1: Neuronal growth regulator 1
