# Supplementary figures and images for: miR-203, fine-tunning neuroinflammation by juggling different components of NF‐κB signaling
Source: J Neuroinflammation. 2022 Apr 12;19:84. doi: 10.1186/s12974-022-02451-9 (PMC9006621; doi:10.1186/s12974-022-02451-9)

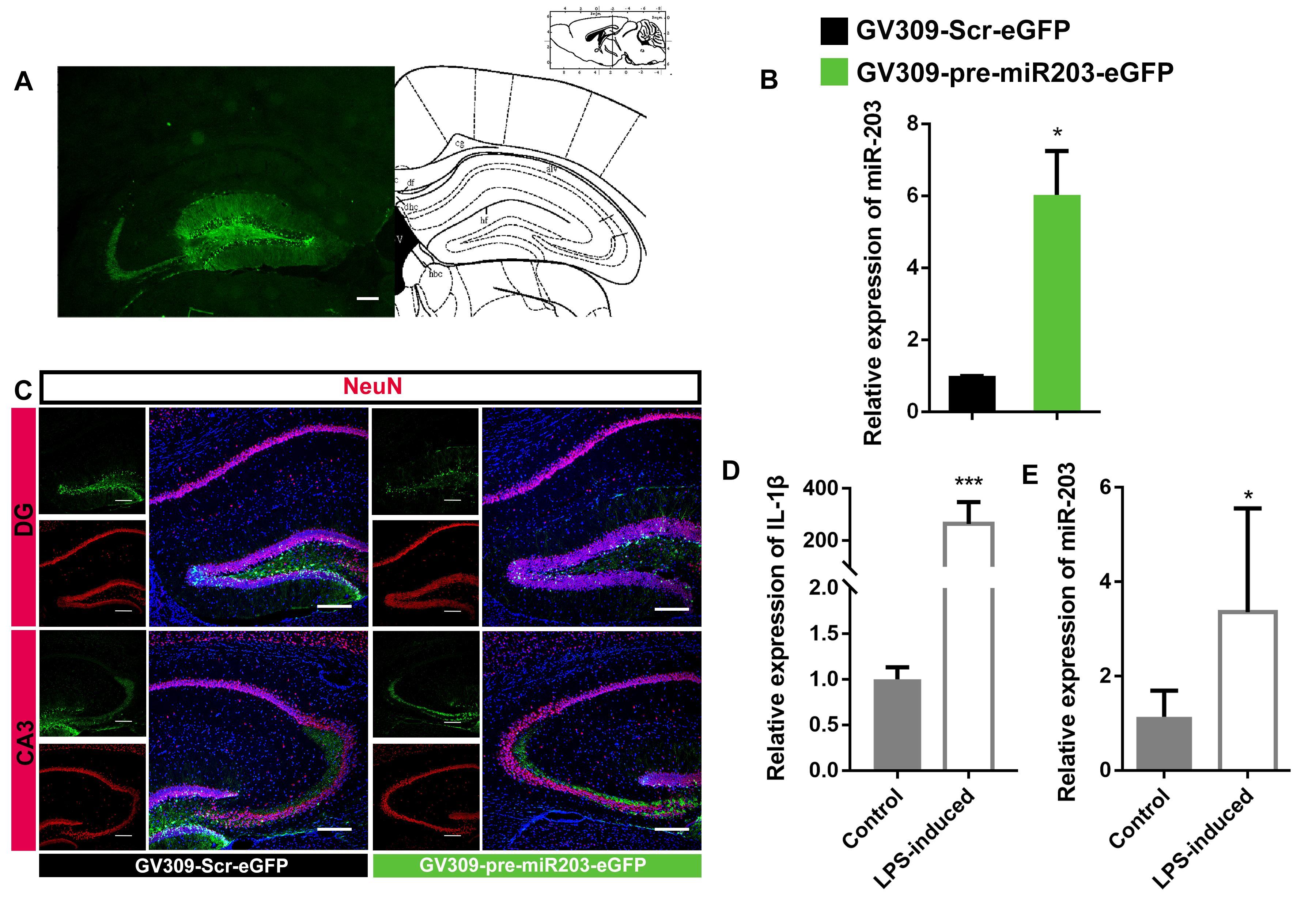

Supplement: Supplementary file 1 — Additional file 1: Figure S1. Stereotactic injection of lentiviral expression vector of GV-309-pre-miR-203-eGFP in the CA3 and DG sub regions of mouse hippocampus. A Bilateral stereotactic injection of GV309-pre-miR-203-eGFP into CA3 (− 2.0 mm at the anterior/posterior axis, ± 2.0 mm at the lateral/medial axis and − 2.0 mm at the dorsal/ventral axis relative to the bregma) and DG (− 1.5 at the anterior/posterior axis, ± 1.5 at the lateral/medial axis and − 2.0 at the dorsal/ventral axis relative to the bregma in mm) in the hippocampus. The scale bar was 200 μm. B Real-time quantitative PCR analysis showing the overexpression of miR-203 in the mouse hippocampus infected with GV309-pre-miR-203-eGFP, compared with controls infected with GV309-scr-eGFP. (n = 3). C Immunofluorescence images showing NeuN positive cells (red) in mouse hippocampus with stereotactic injection of GV309-Scr-eGFP or GV309-pre-miR203-eGFP (green). The lentiviral vector nonselective infected both neuron and nonneuronal cells. The scale bar was 200 μm. D and E Real-time quantitative PCR analysis showing the enrichment of miR-203 expression was significantly elevated in the LPS insulted mouse brains, comparing with the control. (n = 3). Asterisks indicate statistical significance between samples (*, P < 0.05; ***, P < 0.0001). [file 12974_2022_2451_MOESM1_ESM.tif]

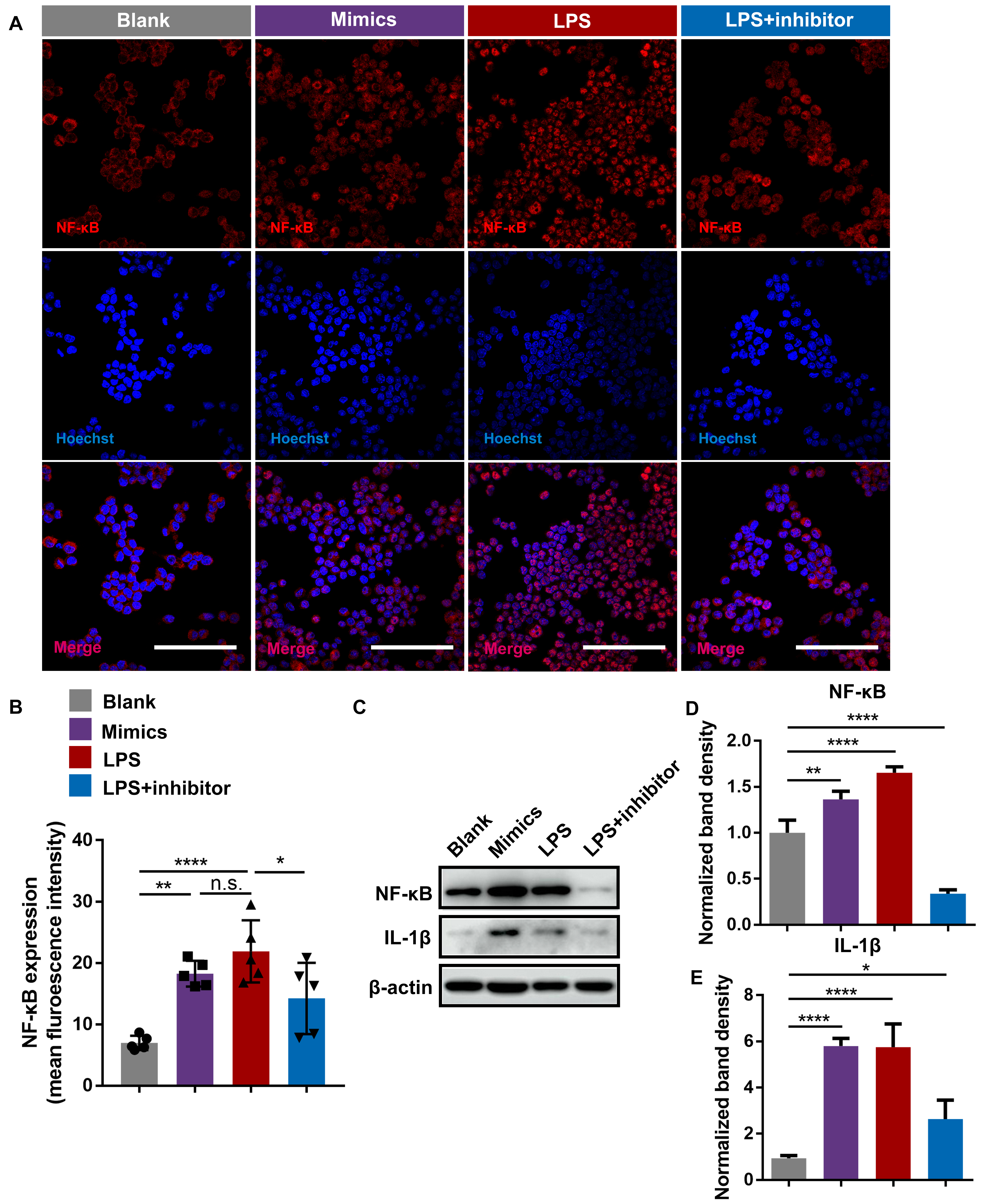

Supplement: Supplementary file 2 — Additional file 2: Figure S2. Overexpression of miRNA-203 promote the nuclear translocation of NF‐κB in RAW 264.7. A The cultured RAW 264.7 cells were transfected with miRNA-203 mimics or miRNA-203 inhibitors. Mock transfections were performed in the blank controls and LPS-induced NF‐κB translocation was used as the positive control. Subcellular localization of endogenous NF‐κB was visualized by immunofluorescent using monoclonal antibody of the p65 subunit of NF‐κB and a Alexa Fluor 647-labeled secondary antibody (red). B The fluorescent intensities of NF‐κB in the nuclei, defined by Hoechst 33342 staining (blue), were quantified and the results of five independent experiments were presented, with the height of columns representing the mean and the error bars showing the S.D. The statistical significance between samples were indicated by asterisks.(one-way ANOVA, Turkey’s post hoc). The scale bars were 150 μm. C Western blots analysis of the protein expression of NF‐κB and IL-1β in RAW264.7 cells transfected with miR-203 mimics, in RAW264.7 cells treated with LPS and in LPS-induced RAW264.7 cells transfected with miR-203 inhibitor. Mock transfection was used as blank control and the expression of β-actin was used as internal control of protein expression. D and E Luminescence-based relative quantifications of protein were expressed as mean ± SD of three biological replicates (one-way ANOVA, Dunnett’s post hoc). Comparing with those in control cells, transfection of miR-203 mimics upregulated the expression of NF‐κB and IL-1β. LPS-induced cells were used as a positive control. Asterisks indicate statistical significance between samples (*, P < 0.05, **, P < 0.01; ****, P < 0.0001) [file 12974_2022_2451_MOESM2_ESM.tif]

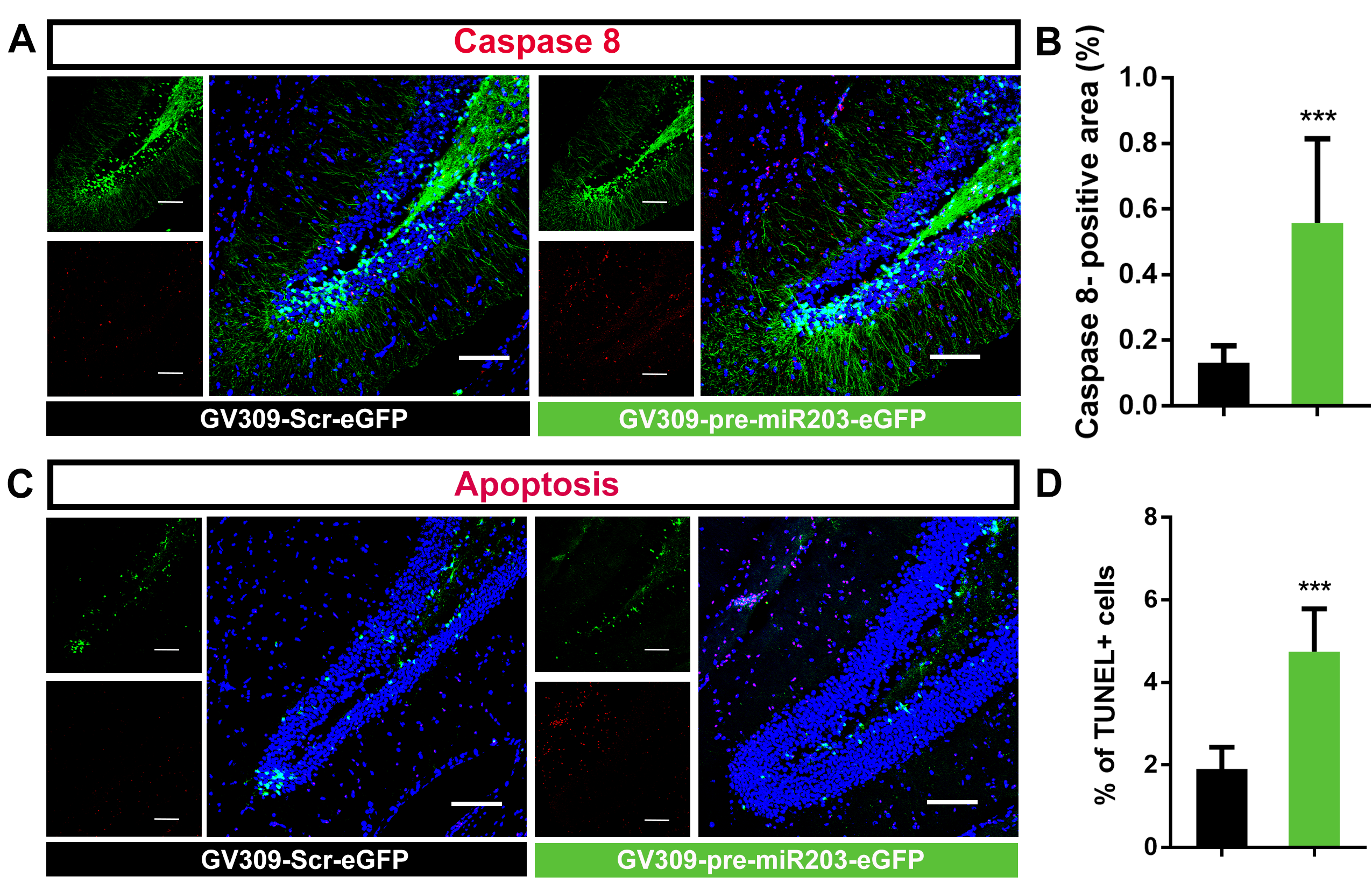

Supplement: Supplementary file 4 — Additional file 4: Figure S4. Overexpression of miRNA-203 induced neuronal cell death in DG regions of mouse hippocampus. A Representative immunofluorescence images showing miR-203 (green) induced elevation of caspase 8 (red) in the hippocampal DG. B Quantification of Caspase 8 positive areas of three consecutive sections consisting of views of bilateral DG of three independent biological replicates were presented as mean ± SD. C Immunofluorescent images of TUNEL assay representing the increased apoptosis in the DG regions of mouse hippocampus with ectopic expression of miR-203. D Quantification of TUNEL positive cells of three consecutive sections consisting of views of bilateral DG of three independent biological replicates were presented as mean ± SD. Asterisks indicate statistical significance between samples (***, P < 0.001) [file 12974_2022_2451_MOESM4_ESM.tif]
